# Prevention starts from the crib: the pediatric point of view on detection of families at high cardiovascular risk

**DOI:** 10.1186/s13052-021-00985-x

**Published:** 2021-03-05

**Authors:** Maria Elena Capra, Cristina Pederiva, Giuseppe Banderali, Giacomo Biasucci

**Affiliations:** 1 Centro Dislipidemie in Età Evolutiva, U.O. Pediatria e Neonatologia, Ospedale G. da Saliceto, Piacenza, Italy; 2 U.O. Clinica Pediatrica, Servizio Clinico Dislipidemie per Lo Studio e La Prevenzione dell’Aterosclerosi in Età Pediatrica, ASST-Santi Paolo e Carlo, Milan, Italy

**Keywords:** Hypercholesterolemia, Screening program, Childhood, Family history, Cardiovascular risk, Paediatric dyslipidaemia

## Abstract

**Background:**

Cardiovascular disease (CVD) is one of the main causes of mortality and morbidity in Italy. Hypercholesterolemia is a modifiable CVD risk factor. The detection and treatment of hypercholesterolemia can modify the natural history of CVD, making CVD risk for affected patients comparable to that of unaffected ones. In this scenario, the detection of families at high cardiovascular risk is the first step of CVD prevention. This multicenter, observational study is aimed at finding an effective and non-invasive screening strategy to detect families at high risk for CVD.

**Methods:**

A survey investigating the knowledge of lipid and CVD issues was distributed to the parents of all infants born at the Neonatology Unit of Piacenza City Hospital and San Paolo Hospital in Milan over a 6 months period. Overall, 554 surveys have been collected.

**Results:**

26.8% newborns had parents who knew their own lipid profile, 40.2% had parents who knew the correct normal blood values of total cholesterol, 37.1% had parents who declared to have first or second degree relatives with lipid disorders, 33.7% had parents who declared to have first or second degree relatives with premature CVD

**Conclusion:**

Collecting a problem-tailored and accurate family history seems to be a good strategy to detect high risk families. Our data suggest that the percentage of adults who are unaware of their lipid profile, with a positive family history for CVD and/or lipid disorders is higher than expected. As a result, even the number of undetected paediatric patients at high cardiovascular risk might be greater than expected.

## Introduction

Cardiovascular disease (CVD) is one of the leading cause of death in adulthood in the United States [1] and also in Italy (www.istat.it). The atherosclerotic process begins even before birth [2] and progresses throughout childhood [3] to adulthood. Post-mortem studies [4] showed that fatty streaks (an accumulation of lipid-filled macrophages within the intima of an artery) are detectable early in life. The Cardiovascular Risk in Young Finns Study [5] showed a positive relationship between cardiovascular risk factors in adolescents and intimal media thickness (IMT), a subclinical marker of atherosclerosis. These studies show that cholesterol levels may be elevated since the first years of life and that untreated hypercholesterolemia in childhood and adolescence is related with higher cardiovascular risk in adulthood. The European Atherosclerosis Society Consensus of Familial Hypercholesterolemia [6] reports remarkable rates of underdiagnosis and undertreatment of elevated blood cholesterol levels; this leads to an increase in morbidity and mortality for CVD. Familial hypercholesterolemia is an autosomal dominant inherited disorder that affects the hepatic metabolism and removal of blood cholesterol particles. Heterozygous familial hypercholesterolemia (from now on referred to as FH) can be considered the most frequent inherited and fatal disease, given that its incidence in the general population ranges from 1:200 to 1:500 [7]. The diagnosis of FH is pretty easy: blood cholesterol test in individuals with a positive family history for CVD and/or lipid disorder is enough to select possible patients; the final diagnosis can be then confirmed by means of a genetic test [7]. Lifelong accumulation of cholesterol in FH patients leads to premature CVD, if left untreated. Conversely, the detection and treatment of hypercholesterolemia can lower the so called “LDL burden”, thus making the cardiovascular risk of the affected individual comparable to the one of the general population [7].

In this scenario, lipid screening in childhood is of utmost importance for CVD prevention, since hypercholesterolemia is one of the main modifiable risk factors for atherosclerosis and CVD [8–10].

As far as we know, cholesterol screening and the management of lipid disorders in childhood have been extensively debated [6] but parents’ knowledge and awareness of the cardiovascular health and lipid disorders in childhood have not been investigated so far. Indeed, the detection of families at high cardiovascular risk is the first step of CVD prevention in childhood, and parents’ awareness and acceptance of their child’s FH condition is a fundamental milestone in the cardiovascular risk prevention. For this reason, we designed this multicenter, observational study aimed at achieving an effective and non-invasive screening strategy to detect families at high risk for CVD and to address them to further specific investigations.

## Materials and methods

**Fig. 1:**
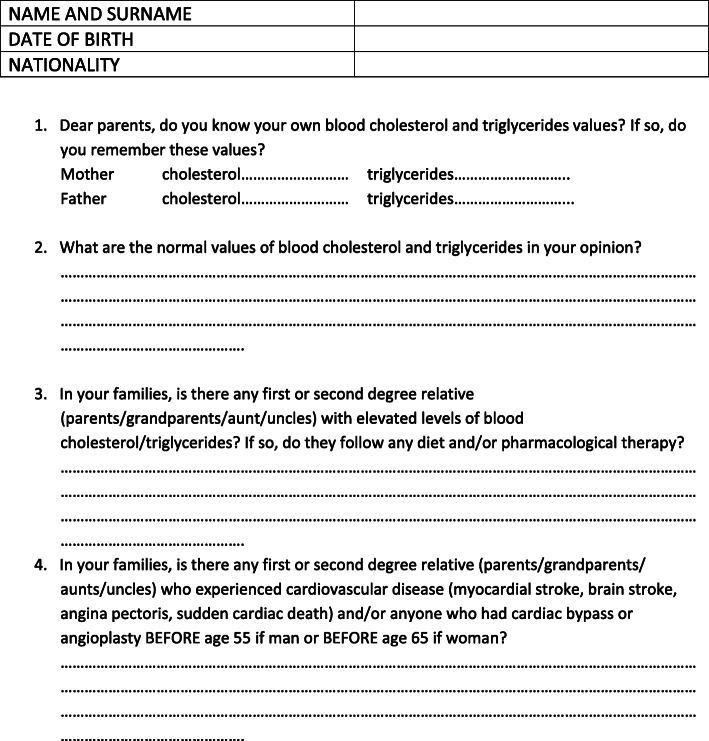
survey investigating the knowledge of lipid and CVD issues distributed to the parents of all infants born at the Neonatology Unit of Piacenza City Hospital and San Paolo Hospital in Milan

**Fig. 2:**
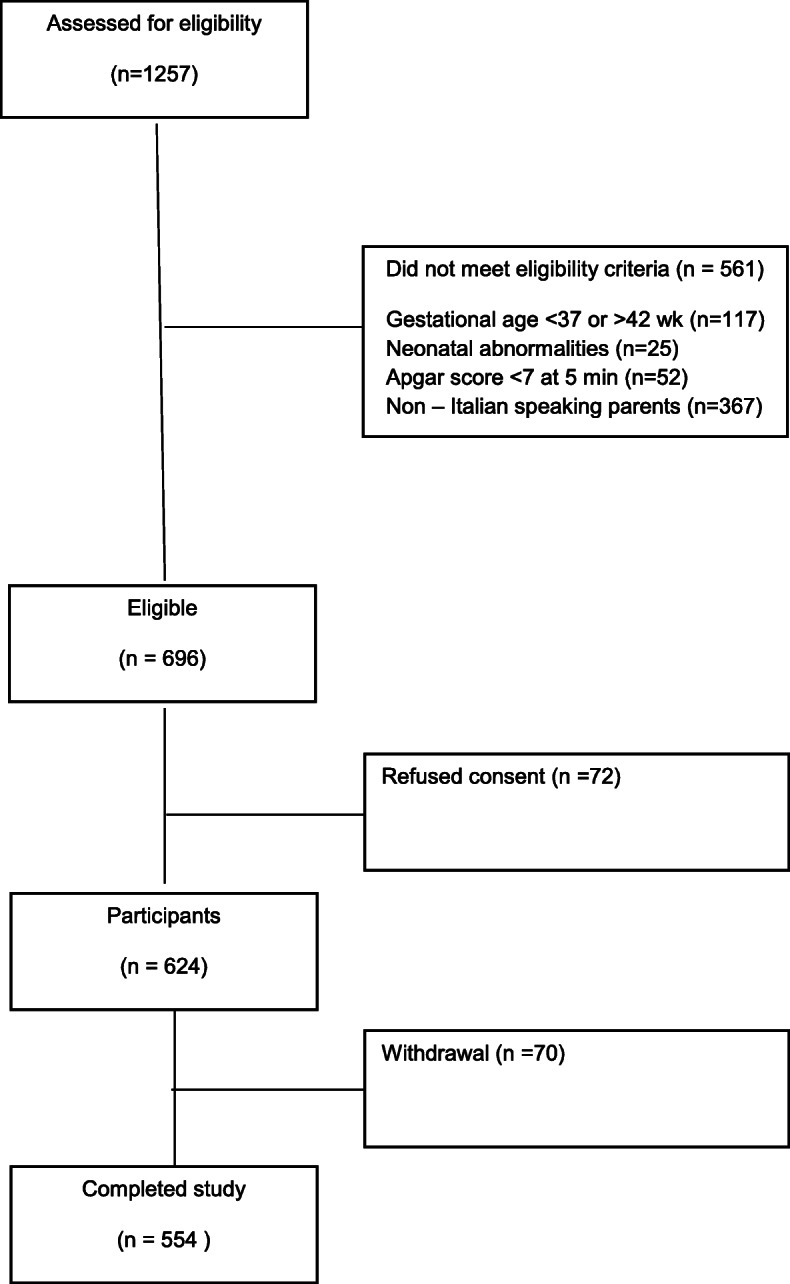
Flow diagram of subject progress throughout the study

A survey investigating the knowledge of lipid and CVD issues was distributed to the parents of all infants born at the Neonatology Unit of Piacenza City Hospital and San Paolo Hospital in Milan over a 6 months period (Fig. 1). The survey was meant to assess parents’ knowledge of their own lipid profile and of the normal lipid profile values, as well as of their own family history for premature CVD and lipid disorders. We considered as normal cholesterol levels below l00 mg/dl and triglycerides below 150 mg/dl [11]. According to international guidelines [8], premature CVD was defined as a cardiovascular event (myocardial stroke, cerebral ischemic stroke) occurring before 55 years and 60 years of age for men and women, respectively. After obtaining an informed consent, skilled physicians working in the aforementioned Neonatology wards helped parents filling in the survey. This one was usually filled out on the day prior to discharge, so as not to interfere with the parents’ emotional impact due to newborn’s birth within the first 24 h of life. Six hundreds couples of newborns’parents in Piacenza City Hospital and 657 couples of newborns’ parents in Milan San Paolo Hospital Neonatology, born in a six-months continuative period (from January 2016 to June 2016), were assessed for eligibility. Eligibility criteria were: term birth, APGAR score above 7 at 5 min, Italian-speaking parents, no neonatal clinical abnormalities. Out of the 600 couples of newborns’ parents assessed for eligibility in Piacenza City Hospital Neonatology Unit, 290 did not meet eligibility criteria; of the remaining 310, 244 completed the study. In Milan San Paolo Hospital Neonatology Unit 657 couples of newborns’ parents were assessed for eligibility: 271 did not meet eligibility criteria, whereas 310 completed the study. The characteristics of the newborns’ population and of their parents are shown in Tables 1 and 2, respectively. The flowchart of subjects’ enrollment is shown in Fig. 2.

**Table 1:** General characteristic of the study population

	All patients	Piacenza City Hospital Neonatology Unit	Milan San Paolo Hospital Neonatology Unit	*p* value between two groups
Number		244	310	
Male (n,%)		130 (53.2%)	160 (51.6%)	
Weight (mean ± sd, g)		Boys 3600 ± 29 g	Boys 3550 ± 35 g	0.45
		Girls 3450 ± 32 g	Girls 3420 ± 37 g	0.47
Gestational age (mean ± sd, weeks)		Boys 39 ± 0.5	Boys 39 ± 0.4	0.53
		Girls 38 ± 0.3	Girls 38 ± 0.3	0.52
		Boys

**Table 2:** Characteristic of the newborn’s parents

	All patients	Piacenza City Hospital Neonatolgy Unit	Milan San Paolo Hospital Neonatology Unit	*p* value between two groups
number		244	310	
Gender		Female 244	Female 310	
		Male 244	Male 310	
Age (mean ± sd, years)		Female 34 ± 0.5	Female 35 ± 1	0.27
		Male 38 ± 1	Male 37 ± 0.6	0.32

## Statistical analysis

The distributional characteristics of each variable, including normality, were assessed by the Kolmogorov-Smirnov test. Descriptive analysis of the general population was performed and subject characteristics were expressed as means ± standard deviation (SD).

To compare qualitative variables, the Chi-square for qualitative variables were used.

## Results

Overall, 554 surveys have been collected: 149 newborns (26.8%) had parents who knew their own lipid profile; 223 newborns (40.2%) had parents who knew the correct normal values of total cholesterol; 206 newborns (37.1%) had parents who declared to have first or second degree relatives with lipid disorders; 187 newborns (33.7%) had parents who declared to have first or second degree relatives with premature CVD. Considering the results divided by center, in Piacenza City Hospital Neonatology Unit and in Milan San Paolo Hospital Neonatology Unit 61(25%) and 88 (28%) newborns had parents who knew their own lipid profile, respectively; 61 (25%) and 162 (52%) newborns had parents who knew the correct normal values of total cholesterol; 88 (36%) and 118 (38%) newborns had parents who declared to have first or second degree relatives with lipid disorders; 73 (30%) and 114 (37%) newborns had parents who declared to have first or second degree relatives with premature CVD.

Considering newborns whose parents had positive family history for lipid disorders (206), 74 (36%) had parents who knew their own lipid profile, 132 (64%) did not know their own lipid profile. Considering newborns whose parents had positive family history for premature CVD (187), 60 (32%) had parents who knew their own lipid profile, 127 (68%) did not know their own lipid profile; 81 newborns had parents with positive family history both for premature CVD and lipid disorders. Among these, 34 newborns (42%) had parents who knew their own lipid profile, whereas 47 (58%) had parents who were unaware. Parents who had never had a lipid screening done, with a positive family history for premature CVD and/or lipid disorders, were addressed to further analysis. Results divided by center are shown in Table 3 and results of all the newborns of the two centers are shown in Graph 1. (Graph 1 title: Surveys results of all patients. Legend: Newborns who had parents answering “yes” to the survey questions are show in blue, newborns who had parents answering “no” to the survey questions are shown in light blue. Data expressed as percentage.).

**Table 3:** Surveys results subdivided by center, expressed as number (percentage)

	All patients	Piacenza City Hospital Neonatology Unit	Milan San Paolo Hospital Neonatology Unit
Analyzed surveys (n)	554	244	310
Newborns whose parents know their own lipid profile	149 (26.8%)	61 (25%)	88 (28%)
Newborns whose parents know correct normal value of total cholesterol	223 (40.2%)	61 (25%)	162 (52%)
Newborns whose parents have first or second degree relatives with lipid disorders	206 (37.1%)	88 (36%)	118 (38%)
Newborns whose parents have first or second degree relatives with premature CVD	187 (34%)	73 (30%)	114 (37%)
Newborns whose parents have first or second degree relatives with BOTH lipid disorders and premature CVD	81 (14%)	37 (15%)	50 (16%)

## Discussion

Hypercholesterolemia is an underdiagnosed and undertreated condition in Italy and in many European Countries [7]. Our study confirms this warning statement, as less of 30% of the interviewed couples of parents are aware of their own lipid profile. Low attention is put on lipid disorders as well, as only 40% of the couples of parents know the correct normal values of lipid profile. The most alarming finding is that, considering those parents who are aware of their positive family history for premature CVD, 4 out of 10 do not know their lipid status and therefore do not know whether they have FH or not. Parents with possible or probable FH have a high percentage of giving birth to a child with the same lipid disorder. Given the utmost importance of a baby’s birth in the parents’ life, we believe this event may help them to become more sensitive to health policies and preventive medicine, in order to protect their newly formed family and to reach the best health outcome. Therefore, unhealthy or irresponsible habits should be eradicated. Moreover, though affected by an undetected hypercholesterolemia, parents might have not yet experienced a cardiovascular event, given their average young age. The diagnosis and treatment of hypercholesterolemia in parents may therefore reduce the morbidity and mortality due to CVD [12]. We believe it is crucial to take care of the whole family at high CVD risk, first by means of a nutritional and life-style intervention, then by adding lipid-lowering drugs, if necessary. International guidelines [8] support lipid screening in children with a positive family history for premature CVD and/or for lipid disorders [13]. In this regard, we believe that the identification of families at high cardiovascular risk is the most important step [14].

We noticed that parents of babies born in San Paolo Hospital in Milan showed a slightly better knowledge of CVD problems compared to those of babies born in Piacenza (Table 1). A possible reason may be that the Milan Pediatric Lipid Disorders Centre has been funded over 15 years ago, whereas the one in Piacenza just 4 years ago. Therefore, pediatricians and general population have been more likely sensitized on lipid and CVD issues in Milan than in Piacenza. However, this difference is not significant and the results from the two centers are overall comparable.

A major limitation of our study is that the population sample is relatively small but, to our knowledge, this is the first study investigating the knowledge and awareness of cardiovascular lipid issues in newborns’ parents. Another limitation is that newborns with non-Italian speaking parents have not been included in this preliminary study. Newborns with non-Italian speaking parents may account up to 25% of the total numbers of newborns in Northern Italy. Moreover, babies born preterm were not included in this study because we preferred to avoid an additional emotionale burden to their parents, even if we know that preterm babies are to be monitored from a CVD point of view [15].

Collecting a problem-tailored and accurate family history seems to be a good strategy to detect high risk families, although the parents’ poor awareness of the problem puts some limits to it. Our data suggest that the percentage of adults who are unaware of their own lipid profile, with a positive family history for CVD and/or lipid disorders is higher than expected. As a result, even the number of undetected paediatric patients at high cardiovascular risk might be greater than expected. In this context, the identification of an effective and reliable screening strategy for CVD in childhood is highly advisable. Moreover, a therapeutic alliance between Pediatricians and General Practitioners seems to be a crucial milestone to detect and treat individuals and families at high CVD risk.

## Conclusion

To our knowledge, the study herein reported is the first one stressing the importance of collecting CVD-oriented family history of the whole family. Moreover, it is the first one to propose a family screening for CVD at birth. The detection of high CVD risk individuals within the first days of life makes it possible to establish early nutritional and behavioral interventions, so as to lower the so called “LDL burden”. Moreover, finding undetected or untreated parents at high CVD risk may interfere with the natural history of CVD, reducing the related mortality and morbidity.

## Abbreviations

CVD: Cardiovascular disease
IMT: Intimal media thickness
FH: Heterozygous familial hypercholesterolemia
SD: Standard deviation
